# “Epidemiology of Health Effects of Radiofrequency Exposure”

**DOI:** 10.1289/ehp.113-a151a

**Published:** 2005-03

**Authors:** Michael Kundi

**Affiliations:** Institute of Environmental Health, Center of Public Health, Medical University of Vienna, Vienna, Austria, E-mail: Michael.Kundi@meduniwien.ac.at

In a recently published review (Kundi et al. 2004) on mobile phone use and cancer, we concluded that

Epidemiological studies that approached reasonable latencies [time period between first exposure and diagnosis] consistently observed elevated risk for the development of neoplastic diseases.

This assessment is distinctly different from the main message of the review from the International Commission for Non-Ionizing Radiation Protection (ICNIRP; Ahlbom et al. 2004). The authors stated that

Results of these studies to date give no consistent or convincing evidence of a causal relation between RF [radiofrequency field] exposure and any adverse health effect.

Although the use of subjective terms is sometimes unavoidable in the context of risk assessment (e.g., to evaluate sufficiency of evidence), the decision whether or not it evidence is “convincing” should be left to the reader. Furthermore, what constitutes consistent evidence or the lack of it is unclear when the scope is as broad as the authors implied in their reference to a “causal relation between RF exposure and any adverse health effect.”

This review of epidemiologic evidence addressed the issue of causation without any consideration of the concept of causation in epidemiology, and it failed in its essential task to assess the possible association between exposure to RF and health. Concerning cancer, Moolgavkar and Luebeck (2003) have shown that agents that increase the growth rate of preneoplastic cells may have a distinctly greater impact on cancer incidence than agents that induce malignant transformation. However, this holds only for agents that act for prolonged periods of time. Regarding the natural history of cancer, a noticeable effect at the population level will only occur many years (and possibly decades) after first contact with the promoting agent. Although Ahlbom et al. (2004) pointed to the insufficient latencies in epidemiologic studies, they did not draw the straightforward conclusion—to assess the relationship between the latencies covered in the studies and their outcome.

Although there is agreement between Ahlbom et al. (2004) and us (Kundi 2004; Kundi et al. 2004) that epidemiologic studies of RF/microwave exposure generally have deficiencies concerning exposure assessment, we must not ignore that the consequence of exposure misclassification is predominantly a bias of risk estimates towards the zero hypothesis.

Another aspect that has contributed to, in our view, the inappropriate assessment of evidence is their view about the end points of the investigations. Among malignancies studied so far, the most heterogeneous group are brain tumors that comprise benign as well as malignant neoplasms with grossly different cellular origin, growth behavior, and fate. Until now no risk factor for brain tumors has firmly been established except ionizing radiation for meningeoma and menigeal sarcoma and less consistently for other brain tumors. Regarding brain tumors of high malignancy, little is known about induction periods and the steps necessary to reach the final invasive state; however, case reports of glioma after sellar irradiation (Simmons and Laws 1998) suggest an average induction period of about 10 years. Therefore, because exposure started too late for an effect during initiation and because proliferation is too fast for an effect on growth rate, brain tumors of highest malignancy must be studied very thoroughly in relation to latency, which was not the case for most of the studies published so far. Disregarding these conditions will strongly dilute any possible effect.

Except for insufficient latency, other sources of possible bias were mentioned by Ahlbom et al. (2004), but again without consideration of the consequences on risk indicators. Ahlbom et al. (2004) stated that

Several of these studies did not follow workers after they left the job of interest (Garland et al. 1990; Grayson 1996; Szmigielski 1996), with the potential for bias if individuals left employment because of health problems that subsequently turned out to be due to cancer....

The presence of this bias in these studies would have reduced the power in the case of no relation between exposure and the likelihood of leaving employment due to early signs of the target disease, or it would have led to a bias of risk estimates in the direction determined by the sign of the correlation between exposure and leaving service. It is quite likely that this correlation is positive because early signs of brain tumors will create problems in radio operators and also in personnel operating and maintaining radar equipment. Hence, the consequence of the bias is either reduction in the precision or inflation of risk estimates.
